# Go Green, Go Social: Exploring the Antecedents of Pro-Environmental Behaviors in Social Networking Sites beyond Norm Activation Theory

**DOI:** 10.3390/ijerph192114265

**Published:** 2022-11-01

**Authors:** Chia-Ying Li, Yu-Hui Fang

**Affiliations:** 1 Department of Business Administration, National Taichung University of Science and Technology, Taichung 404, Taiwan; 2 Department of Accounting, Tamkang University, No. 151, Ying-Chuan Rd., Tamsuip, New Taipei City 25137, Taiwan

**Keywords:** norm activation theory, pro-environmental behaviors, egocentric tactician model, guilt, social stressor

## Abstract

The paucity of environmental resources and the threatening warning of global climate change have led to increasing research on environmental issues [e.g., pro-environmental behaviors (PEBs)]. Although norm activation theory (NAT) is a well-recognized theory for approaching PEBs, existing works appear insufficient to explain PEB in the context of social networking sites (SNSs) without taking contextual, emotional, and social factors into account. Grounded in the egocentric tactician model (ETM), NAT, along with the notions of guilt and social stressors, this study integrates a new ETM path, a supplemented emotional path, alongside the conventional NAT path to achieve a more complete picture of what are crucial determinants of PEBs in the context of SNSs. Social stressors positively moderate the emotional path. Data collected from 897 Facebook users confirm all of our proposed hypotheses. Results indicate that beyond the traditional NAT path, the new ETM path and the emotional path add values to illustrate PEBs on SNSs, and new constructs of self-influence on SNSs (SIS) and guilt remarkably drive PEBs alongside personal norms. Implications for theory and practice are discussed, and guidelines for future research are identified.

## 1. Introduction

The paucity of environmental resources, the hastening pollution of the earth, and the threatening warning of global climate change have led to increasing research on environmental issues. Among them, pro-environmental behaviors ([PEBs], behaviors that help, rather than hurt, the environment [1]) is a popular environmental issue because PEBs, more or less, can prevent the environment from being acceleratingly destroyed. The constructive value of PEBs has motivated diverse research streams to provide insights into the determinants of PEBs. For example, some studies [2] identified the trends and patterns in PEB research, some [3] discussed the means to measure PEB, and others focused on organizational constructs (e.g., corporate social responsibility [4,5] and green transformational leadership [6]. Although previous works on PEBs have applied the theory of planned behavior (TPB) [7] and the view of positive emotion (e.g., positive and self-transcendent emotions) [8], norm activation theory (NAT) is a more well-recognized theory to approach PEB [9,10]. NAT [11] postulates that people are aware of the outcomes of no further actions (awareness of consequences) and their responsibilities for environment protection (ascription of responsibility (Note that both awareness of consequences and ascription of responsibility are called activators in NAT literature)) upon receiving eco-messages, which may motivate their personal norms and the sequent PEB to benefit the environment (namely a NAT path). However, as researchers have remarked, a crucial means to advocate PEBs is to disperse eco-messages [12]. It is difficult to encourage PEBs if choosing an improper communication channel to disseminate eco-messages [12]. With social media, diffusing eco-messages on social networking sites (SNSs) apparently may be an efficient way to promote PEB due to the reach of SNSs outperforming other channels. Although NAT theorizes the whole mental process, and sounds intuitively rational, it does not take emotional concerns nor a social perspective pertaining to the applied context (i.e., SNSs) into account. As Aristotle once said, human beings are emotional and social animals. This study suggests that taking emotional, social, and negative factors into consideration would present a more comprehensive view to explore PEB in the investigated context.

In addition, recent information system (IS) scholars [13] have called for more attention to be paid to challenges pertaining to climate change because IS research has relatively lagged behind other fields in this direction. These phenomena, collectively, raise questions: (1) Is NAT and its related factors (e.g., awareness of consequences, ascription of responsibility, and personal norms) sufficient to explain PEB in the context of SNSs? and (2) Taking SNSs into account, will other theories and factors add value to clarify PEB? Put differently, what are critical determining factors of PEB in SNSs? Since existing works on the above issues appear insufficient, this study aspires to integrate multidisciplinary theories to address these questions.

In view of these research gaps and our first question above, this study suggests guilt and social stressors to be valuable constructs to advance the NAT path. First, as an unpleasant emotional state [14], guilt represents a negative and emotional perspective. Pioneering researchers have identified the positive effect of guilt on pro-social behavior (knowledge sharing [14]) and eco-friendly responses [15]. Likewise, alongside personal norms, guilt may provoke PEBs and form an extra emotional path to the traditional NAT path. Second, as Chaiken and Eagly [16] claimed, the true stimuli of information received and subsequent responses may vary depending on receivers’ experience and features. Since a social stressor, characterizing the burden of social overload in the context of SNSs [17], can provoke certain negative effects (e.g., psychological and behavioral strain, dissatisfaction, SNS exhaustion [18,19]), it may serve as a moderator to regulate the emotional path. To obtain an advanced understanding and a systematical analysis of the NAT path, this study plans to investigate the unknown moderating effect of a social stressor.

Furthermore, since our investigated context is SNSs, it is notable that everyone could become influencers to change others and show off themselves in the environment of SNSs. Upon this backdrop and the second question above, first, this study focuses on two types of PEBs—private-sphere PEB and public-sphere PEB (hereafter called “private PEB” and “public PEB”). Private PEB focuses more on one’s daily pro-environmental activities (e.g., recycling), whereas public PEB is related to environmental citizenship (e.g., supporting pro-environmental policies and activities [20]). Most studies in this field have merely looked at either the private PEB or both PEBs in the physical context [21,22], ignoring the superior influence of information diffusion available in SNSs [23]. Since both PEBs are relatively easier to be observed in the context of SNSs (e.g., through the check-in function and an update in one’s news-feed) than in physical settings [23], it is time to take both PEBs into account.

Second, to investigate our second question, we draw upon an egocentric tactician model (ETM) [24] to scrutinize what motivates people to manipulate their influence to affect their connections (e.g., contacts, friends, or fans [25]) through PEBs in SNSs. According to ETM, we propose that individuals’ self-motives (e.g., self-enhancement) and social motives (e.g., social-enhancement and response efficacy) could activate their self-knowledge (self-influence on SNSs; SIS), which in turn promotes their PEBs on SNSs (namely a ETM path). The proposed ETM path and the broader view of PEBs could complement the insufficiency of existing NAT literature in illuminating the determinants of PEBs in the context of SNSs.

Overall, grounded in the ETM, NAT, along with the notions of guilt and a social stressor, this study integrates a new ETM path, a supplemented emotional path, alongside the conventional NAT path to achieve a more complete picture of what are crucial determinants of PEBs in the context of SNSs. Social stressors, as a moderator in the emotional path, helps in the reaching of an enhanced interpretation of the proposed relationships. This study contributes to current literature in four ways. First, by exploring and empirically testing the two proposed paths towards PEBs, it provides evidence and support on how SNSs serves as an efficient channel to diffuse eco-messages and promote PEBs. This investigation also responds to Pan et al.’s [13] call for greater IS research efforts. Second, by incorporating social factors (e.g., social stressor, social motive), it offers better understanding of a hitherto unexplained issue—how these social concerns play when promoting PEBs in an emerging social channel. Third, by extending ETM to merge with NAT, it adds value to existing literature on PEB by introducing new constructs (i.e., a self-motive, social-motives, and SIS). The boundary of ETM is currently expanded to the field of PEB and eco-message diffusion on SNSs. Meanwhile, this investigation fills the research gap which Fang et al. [23] have called for continuous attention of SNS influence in various phenomena. Fourth, by taking social stressors into account, it provides a more insightful view of the added emotional path and thus contributes to the traditional NAT path from a negative perspective.

## 2. Theoretical Background

### 2.1. Eco-Message Diffusion and Pro-Environmental Behavior in Social Networking Sites

Diffusion of eco- or green messages is a means toward raising awareness among people to environmental concerns [26], which may change people’s environmental attitudes and guide their PEBs (e.g., selection of eco-friendly products [12]). Scholars have attributed the failure of eco-messages in changing people to the unsuitable selection of communication channels, ineffective communication, less attention-attracting, and low-credible information sources [12]. In this respect, dispersing eco-messages through SNSs paves an efficient way to reaching and influencing people because of the four capabilities of SNSs in overcoming the aforementioned barriers. First, SNSs (e.g., Facebook) have “a richer, more intimate hoard of information about its citizens than any nation has ever had” [27] (p. 36). Second, trustworthy sources of information on SNSs (e.g., from people’s connections—contacts, friends, or fans) enable eco-messages to draw more attentions and increase their credibility [25]. Third, by affording a variety of kinds of content (e.g., text, sound, photos, films, files [14,28]), SNSs enable information richness. Fourth, by building connections among diverse audiences—individuals, small and large groups [14], SNSs enable relatively faster and wider information diffusion than other physical settings [28]. Previous works [23,25] have consistently recognized the speedy information diffusion on SNSs and thus supported the inference to eco-message diffusion on SNSs. Overall, these observations explicitly justify the superiority of SNSs over other contexts in distributing eco-messages, which might provide a better opportunity to encourage PEBs and ultimately help the global environment.

As we mentioned earlier, both private and public PEBs are relatively more tractable specifically in the context of SNSs than traditional environments because of the available functions embedded in SNSs (e.g., check-in function, updates in one’s news-feed, notifications receiving from followed and favored events or campaigns [28]). However, despite the wide penetration of SNSs, limited research attention has hitherto been devoted to the issue regarding how eco-message distributing on SNSs instigates people’s PEBs. In addition, since past works [14,29,30] have confirmed the motivational force of emotion (e.g., guilt) to take constructive actions (e.g., pro-social behavior [14], eco-friendly responses [15], ethical consumption, and recycle behavior [30,31,32]), guilt may play a role to encourage PEBs in our investigated context. This study thereupon constitutes a first attempt to explore this matter by integrating theories of NAT, guilt, and social stressors along with ETM, and empirically test them. The next section discusses the applied theories and notions.

### 2.2. Norm Activation Theory

The widely used theory that explicitly explains PEBs is norm activation theory (NAT). NAT was initially developed to understand diverse types of prosocial or altruistic behaviors [11]. It depicts the relationships among activators, personal norms, and behaviors. According to NAT, norm activation is about a process in which people develop self-expectations about prosocial behaviors. These self-expectations, namely personal norms, denote feelings of moral obligation to execute particular actions [33]. Personal norms will be activated and then prosocial behaviors will be endorsed when people recognize the negative consequences of not helping (awareness of consequences) and attribute responsibility for these consequences to themselves (ascription of responsibility) [10]. As abovementioned, pioneering studies in environmental psychology have empirically confirmed the applicability of NAT in predicting general PEB [9] and treat PEB as a special kind of prosocial behavior to benefit others without direct reciprocity [10]. Accordingly, this study uses NAT as a point of departure to explore PEB-related issues in social networking sites (SNSs) and to test whether the activator-norm-behavior linkage remains powerful in the case of SNSs.

Notably, when applying NAT to the context of SNSs, it is uncertain whether existing NAT factors (e.g., awareness of consequences, ascription of responsibility, and personal norms) would be sufficient to predict PEBs on SNSs because NAT conventionally focuses on factors in terms of the physical context. It is notable that certain social, emotional, and negative factors should not be ignored when studying SNSs. Therefore, this study begins to fill the previous gap in our knowledge by integrating a notion of guilt and theory of social stressors into our model because it could threaten user participation in SNSs and result in negative consequences [18]. The next section describes the theory and related social factors.

### 2.3. Theory of Social Stress

Generally, people can enjoy some positive benefits through the use of SNSs, such as establishing close social relations, expanding social circles, and obtaining social supports [34]. Meanwhile, possible drawbacks, however, may lead people to the dark side of social stressors because various social overloads occur due to an excessive use of SNSs [18,35]. Indeed, the issue of social stressors has attracted increased attention. A social stressor—derived from social action, information, and communication overloads—has been identified as the latest and most powerful kind of stressor inducing psychological and behavioral strain, especially in the use of individuals’ social networks [17]. According to Maier [17], social action overload represents a negative feeling when a user perceives he/she is giving too much social support to other individuals in his/her virtual social network. Social information overload describes a negative perception when a user receives too much information from SNSs to deal with, while social communication overload is a negative feeling that comes about when a user perceives his/her SNS interaction to be undesirable. Overall, these three facets of social overload jointly represent social stressors embedded in the context of SNSs.

Notwithstanding, pioneering work has indicated the prominent influence of social stressors in the case of SNSs (e.g., SNS fatigue, dissatisfaction, SNS exhaustion, and SNS discontinuance [18,19,36,37,38]). However, no previous work has applied theory of social stress to explore the issue of eco-messages and PEBs on SNSs. Indeed, previous PEB studies largely focus on its antecedents using NAT from a positive viewpoint. However, beyond positive influence, eco-messages received on SNSs may impose a burden of social stressor on people that intensifies their negative thoughts and thus alters their subsequent actions. Hence, in order to comprehensively understand the influence of eco-messages on PEBs in SNSs, it is reasonable to incorporate both positive and negative views to broaden existing research on NAT and PEBs.

### 2.4. Guilt

Guilt is a negative emotional state deriving from potential undesirable outcomes due to one’s actions, inaction, or circumstances [32]. As a self-conscious moral emotion [39,40], guilt deals with “some past behavior that is inconsistent with the set of internalized standards—often, but not necessarily, moral in nature” ([41], p. 103). Notwithstanding that guilt is an aversive emotion, its motivational force to take constructive actions has been consistently remarked on [14,30]. Typically, people experience guilt when they are aware that they should have behaved differently [15] to prevent the mis-happenings. They, thereupon, are motivated to take reparative actions (e.g., confessions, apologies, making amends) to rectify their past behaviors and ease the unwanted results [14,39,40]. Hence, scholars conclude guilt as a protective factor to do good and avoid doing bad [40]. In essence, the motivational effect of guilt has been consistently found on interpersonal communication [14], advertising practices [15], marketing campaigns [39], and charity campaigns [42]. Recent studies have increasingly applied guilt to pro-environmental issues (e.g., ethical consumption and recycle behavior [30,31,32]) and confirmed its significant role in promoting altruistic behavior and pro-social behavior [29]. Surprisingly, to our knowledge no studies to date have incorporated the notion of guilt with NAT to investigate PEBs in the context of SNSs, thus leaving a gap to be bridged. 

### 2.5. Egocentric Tactician Model

Although theories of NAT, guilt, and social stressors offer the negative and emotional view in illuminating the antecedents of PEBs on SNSs, they are not sufficient to comprehensively explain the matter due to the lack of positive consideration. Given that people are inherently self-oriented and tend to view themselves favorably [43], it is beneficial to take both positive and negative views into account to shed more light on PEBs, indicating a need for new theories. Indeed, the Egocentric Tactician Model (ETM) might be such a theory because it describes how self-concept changes people’s thoughts about their social surroundings and those who dwell around them [24]. Although the issue regarding how self-view guides one’s social thinking has been discussed in previous works, in part [43], they fall short of an overarching picture of it. ETM provides a relatively holistic framework in this direction.

Against this background, our extension of ETM to the matter of PEBs in the context of SNSs accompanies the negative view to achieve a more comprehensive model. In essence, contrary to the negative view of NAT, guilt, and social stressors, ETM hinges on a positive view of cognitive structures and asserts that people aspire to judge themselves positively [44]. According to Sedikides et al. [24], such cognitive structures incorporate: (1) one’s positive self-knowledge; (2) the knowledge (e.g., one’s beliefs and experiences) concerning one’s social realm and its dwellers; and (3) how both the knowledge and its configuration, along with external factors, determine how information processed through those structures is retrieved and ultimately exert a substantial influence on one’s social thoughts and behaviors. Specifically, ETM considers self-enhancement as one crucial motive to fulfill the self-perception goal. Beyond its egocentric nature, ETM theorizes that social thinking is not only directed by diverse self-motives but also coordinated to gratify those motives due to its tactical spirit. These observations suggest the motive-self-behavior scheme of ETM as well as the need for including more external variables alongside the self-motive. Since ETM has neither specified particular constructs for the scheme nor empirically investigated the model, this leaves certain research gaps to be closed. Accordingly, this study incorporates self-enhancement as a self-motive construct, self-influence on SNSs (SIS) as a construct of the self-knowledge, and two PEBs as behavioral constructs. Taking the context of SNSs into consideration, this study also proposes two social-motives (response efficacy and social-enhancement) as additional motive factors to move forward the application of ETM to SNSs and add value to ETM literature in this direction.

## 3. Research Model and Hypotheses Development

**Figure 1:**
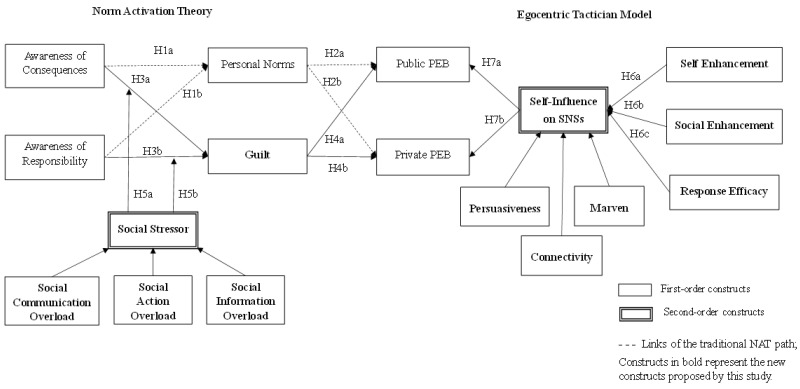
Research Model.

This study incorporates ETM and NAT along with the notions of guilt and social stressors to devise a more complete model for illustrating PEBs on SNSs. Initially, in line with PEB literature [11], this study treats awareness of eco-messages as activators of norm, which are manifested by awareness of consequences and ascription of responsibility. According to NAT, activators have been consistently identified as key to activating one’s obligation to PEBs [10,45]. When applied to the context of SNSs, it is possible for the same activators to arouse a negative emotion of guilt while taking social, negative, and emotional consideration into account. Analogously, a social stressor may regulate the relationships asserted by NAT. It is rational to expect activators, as a starting point, to activate not only personal norms (positive stress) but also guilt (negative stress) on SNSs. Departing from awareness of eco-messages, our proposed model incorporates two paths to PEBs in terms of negative and positive considerations (Figure 1). The left part of the model (called NAT model) illustrates the conventional NAT path (i.e., activator-norm-behavior path) alongside a negative emotional path (i.e., the activator-guilt-behavior path). A social stressor moderates the activator-guilt relationships embedded in the emotional path. The right part of the model, mainly based on ETM [24], puts forth a positive ETM path (i.e., motive-self-behavior path). Beyond the traditional NAT view, the new, supplementary ETM path may shed further light on PEBs in the context of SNSs. The next subsections describe the related literature and support for the model. 

### 3.1. The Norm Activation Theory Path

The original NAT path (see the dotted links in Figure 1) is primarily based on the traditional NAT and includes essential constructs: awareness of consequences, ascription of responsibility, personal norms, and PEBs. Awareness of consequences reflects one’s awareness of possible consequences for others if one does not act pro-socially [46]. Ascription of responsibility represents the acknowledgement of one’s responsibility for the possible outcomes of not acting pro-socially [46]. Personal norms denote a moral obligation to enact or avoid doing specific behaviors [33] and are about one’s self-expectations for a particular action that derives from individual norms and values [11]. Finally, PEBs focus on individual actions that bring about reduced resource use and environmental influence, which can be attained by both undertaking behaviors that help the environment and demoting behaviors that hurt the environment [47]. As per NAT, PEBs are a function of personal norms which are initiated by two activators—awareness of consequences and ascription of responsibility [11]. Since the relationships between these key constructs have received considerable support from previous studies [9,11], brief descriptions are discussed below with respect to the activator-norm-behavior relationships.

#### 3.1.1. Activators and Personal Norms

As abovementioned, awareness of eco-messages manifests in awareness of consequences and ascription of responsibility, serving as activators of personal norms in this study. The positive relationships between awareness of consequences and ascription of responsibility and personal norm are well-established in existing literature in NAT and PEBs [11,45]. Generally, it is awkward for people to generate a strong obligation to take an action without being aware of the negative consequence and of the responsibility of their inactions. That is, when people are attentive to environmental needs and realize that their actions may help to achieve environmental needs, their personal norms are more inclined to be provoked and thus they dedicate themselves to constructive actions (e.g., PEBs [9,45]). Hence, the above arguments put forth the first set of hypotheses:

**H1a:**  *Awareness of consequences is positively related to personal norms*.

**H1b:**  *Ascription of responsibility is positively related to personal norms.*

#### 3.1.2. Personal Norms and Behaviors (Private and Public PEBs)

PEBs, a particular type of prosocial behavior, have been recognized as resultant behaviors of personal norms [9,11]. Following the most common PEB classification [20], this study applies two types of PEBs to the context of SNSs—private and public PEBs. Private PEB embraces more daily activities, including “the purchase, use, and disposal of personal and household products that have environmental impact” [20] (pp. 409–410). Alternatively, public PEB corresponds to environmental citizenship and the support of pro-environmental policies [20]. Seemingly, in traditional physical settings, public PEB requires more effort and time to perform than that of the private PEB [21]. However, when applied to the case of SNSs, public PEB is not only easier to be implemented but also to be observed by others because of the speedy information transmission available in SNSs [23]. In addition, scholars have confirmed that both PEBs are of a heterogeneous nature and have their unique influence on individuals and society, directly or indirectly [22]. However, relatively few studies have examined public PEBs on SNSs. Altogether, these observations denote the need to explore whether or not the relationships between personal norms and the two PEBs remain still, even in the context of SNSs. According to NAT, when people sense a feeling of moral obligation to behave pro-socially, they will be inspired to perform certain PEBs in keeping with their value systems [48]. The positive relationship between personal norm and various types of PEBs (e.g., recycling, energy conservation) has been well supported [45,49]. Hence, the second set of hypotheses are proposed as follows:

**H2a:**  *Personal norm is positively related to public PEB.*

**H2b:**  *Personal norm is positively related to private PEB.*

### 3.2. Guilt and the Emotional Path

Beyond the original NAT path, the notion of guilt and its associations with activators and PEBs build an extra emotional path to complete the left-part of the NAT model. This postulation is reasonable because guilt has been highlighted as an imperative factor to effectively change human behaviors in social campaigns (e.g., marketing and environmental campaigns [32]), independent of personal norms. Consequently, the emotional path (the activator-guilt-behavior path) complements the traditional NAT path (i.e., the activator-norm-behavior path) to move forward the NAT literature. The next subsections discuss research hypotheses of the emotional path pertaining to activators and resultant behaviors (PEBs) of guilt. 

#### 3.2.1. Activators and Guilt

Activators are manifested by awareness of consequences and ascription of responsibility in this study. Awareness of consequences pertains to one’s mindfulness about detrimental consequences when not engaging in pro-social behaviors [11]. In a sense, awareness of environmental problems may bring about negative emotions (e.g., guilt) if these problems are not settled [50]. Han et al.’s [51] work also supports the positive association between awareness of consequences and guilt. Correspondingly, upon receiving eco-messages on SNSs, if people realize environmental problems and harmful outcomes, they may feel guilty about not undertaking environmentally-friendly actions. Thus, this observation establishes the link between awareness of consequences and guilt. 

Ascription of responsibility describes the sense of self-responsibility for the unfavorable outcomes of not behaving pro-socially [46]. Although ascription of responsibility has been typically linked to personal norms [9,45], its link with guilt does have its theoretical backing. As Baek and Yoon [15] stated, guilt is a self-conscious moral emotion. Guilt is easily excited after self-evaluating past behaviors or inactions concerning environmentalism [52]. If people feel responsibility for the harmful consequences resulted from not executing some actions, they may perceive the self-conscious emotion of guilt. In line with literature development, ascription of responsibility is expected to directly influence guilt in this study. Overall, two hypotheses related to activators and guilt are put forth:

**H3a:**  *Awareness of consequences is positively related to guilt.*

**H3b:**  *Ascription of responsibility is positively related to guilt.*

#### 3.2.2. Guilt and PEBs

Although guilt is a negative emotion, previous literature has noticed its constructive effect on contrasting motivations and actions (e.g., pro-social behaviors, knowledge sharing [14,52]). Indeed, guilt, albeit generated from the ascribed responsibility for previous inappropriate behaviors, can work as an adaptive function by shifting people’s motivational focus to their underperforming area, and make reparation for counteracting detrimental consequences [14]. As such, guilt has been known as a pro-social emotion [51], which serves as a key driver of pro-environmental decisions [53]. In addition, as scholars [40] suggested, the experience of guilt may lead to more attempts to reduce one’s uncomfortableness accompanying corrective actions and proactive pursuits. It is plausible that upon receiving eco-messages on SNSs, when people feel guilty about their inactions and improper behaviors, they are more likely to enact environmentally-friendly behaviors (private PEB) and endorse pro-environmental campaigns (public PEB) as compensation. Hence, the two hypotheses regarding guilt and PEBs are proposed as follows:

**H4a:**  *Guilt is positively related to public PEB.*

**H4b:**  *Guilt is positively related to private PEB.*

### 3.3. Social Stressor as a Moderator of the Activator-Guilt Relationships

Social stressors are demands, circumstances, situations, or episodes in a social environment that produce stress [54]. A social stressor symbolizes the social factor that generates stress, in which the concept of overload is commonly recognized as a representative stressor [17]. Historically, overload reflects one’s subjective assessment and judgment of the amount of objects or people that surpass one’s capabilities to deal with [55]. Three overload factors—social action overload, social information overload, and social communication overload—are identified by Maier [17] to manifest social stressors in relation to the context of SNSs. Specifically, social action overload refers to a negative perception when one obtains excessive amounts of social support requests and perceives one is offering immoderate social support to other people in one’s SNS. Social information overload refers to a negative perception when one is exposed to excessive amounts of information engendered on SNSs, which goes beyond one’s processing ability [56]. Social communication overload is about a negative perception when communication requests from diverse channels within one’s SNS (e.g., emails, instant messaging, and news feeds) outrun one’s communication capacities [57]. Obviously, when all of these three aspects of overload are taken into consideration, they can best exhibit the concept of social stressor.

Apparently, a social stressor is a context-specific construct related to various overloads of SNSs that generates people’s psychological stress and negative thoughts [17,37]. According to Baron & Kenny [58], contextual factors frequently act as moderator variables because they can systematically transform the associations between predictors and target variables. Analogously, a social stressor may be such a moderator to intervene in the relationships between activators and guilt alongside the emotional path. Explicitly, in the case of eco-message diffusion on SNSs, the social stressor generated from the use of SNSs may boost the strength of the relationships between activators (i.e., awareness of consequences and ascription of responsibility) and guilt. This postulation is logical because both notions of social stressors and activators stand for negative considerations toward SNSs, in which their interactions may aggravate their influence on guilt. In a sense, people experience high overloads, overburdening, and stress from the use of SNSs, resulting in their psychological change and the raising of their negative thoughts [35]. Upon receiving eco-messages on SNSs, these negative thoughts may regulate their attention to outweigh the impacts of unfavorable consequences and responsibilities for their inactions, thus exacerbating their negative feelings of guilt. Concisely, the salience of awareness of consequences and ascription of responsibility on guilt may change under different levels of social stressor. Accordingly, it is plausible to infer that people with high social stressors will feel more guilty when they are aware of the harmful consequences of not helping and when they notice their responsibility for these detrimental results. The moderating hypotheses are proposed as follows:

**H5a:**  *Social stressor positively moderates the relationship between a* *wareness of consequences and guilt.*

**H5b:**  *Social stressor positively moderates the relationship between ascription of responsibility and guilt.*

### 3.4. The Egocentric Tactician Model Path

The right-part of the model (namely the ETM path) is built on the ETM [24] and involves applicable constructs corresponding to the motive-self-behavior scheme. First, this study incorporates self-motive and the coordinated social-motives to represent the motive factors because both self-motive and social-motive have been identified as egoistic motives [59] and are consistent with the egocentric view of ETM. Explicitly, the self-motive is manifested by self-enhancement [24], whereas social-motives are manifested by social-enhancement and response efficacy in this study. Then, the self-concept is manifested by the construct of self-influence on SNSs (SIS) in this study because SIS portrays self-knowledge about one’s own influence attainable in the context of SNSs [23]. Finally, according to ETM [24], people’s behaviors (e.g., PEBs) may be activated by their positive self-influence (SIS), which is motivated by certain motives (self-enhancement, response efficacy, and social-enhancement). Thereupon, these assertions justify four related hypotheses, which altogether construct the ETM path. The next section explicitly addresses the hypothesis development.

#### 3.4.1. Motives and Self-Influence on SNSs

To initiate the ETM path, this study positions SIS as the focal self-view in the investigated context because it symbolizes one’s positive thought about self-influence regarding one’s social milieu and those who one hangs around [24]. Besides, the influence attributes of SIS are more noticeable in the context of SNSs than they are in the traditional offline contexts [23]. Based on the pioneering work on information diffusion [23,60], maven, persuasiveness, and connectivity are identified as three dimensions of SIS that can regulate information diffusion and direct people’s action. Specifically, maven characterizes an individual who possesses broad knowledge about a particular issue, initiates deep discussions with others, and responds to requests from others for further information. Hence, mavens hold considerable expertise in a specific theme across multiple areas. Persuasiveness refers to one’s ability to convince others to agree with a suggestion. Persuasive people are good at conveying their issue positions from multiple aspects and hence effectively persuade others to constructively see their view of a matter. As such, persuasive people can be regarded as argumentative and effective influencers. Connectivity reflects one’s ability to influencing others by bridging diverse individuals and groups. Typically, good-networked people are competent in connecting people, may lessen physical and social distances between people, and are prone to be connectors between distinct groups (e.g., their contacts and contacts’ contacts [60]). Indeed, as Fang et al. [23] suggested, SNSs serve as an effective platform to keep track of one’s contacts and leverage one’s expertise to influence them. Taking these three dimensions into consideration can properly characterize not only self-influence in the context of SNSs but also the self-view of the ETM.

According to ETM [24], self-influence thought is a function of egoistic motives, i.e., self-motive and social-motives [59]. Self-enhancement corresponds to the self-motive in this study due to its self-evaluation nature [24,61]. Self-enhancement refers to the interest and craving for advancing oneself or boosting the positivity of the self [62]. Literature on self-enhancement contends that individuals desire to feel positive about themselves and strive for others’ approval [63]. As Jones [64] claimed, individuals “want to increase, confirm, and maintain personal satisfaction, worth, and effectiveness” [64] (p. 186). Since the self-enhancement motive strengthens one’s tendency to interpret events in a way that maximizes one’s positive self-attributes, it can energize positive thinking, further involvement, and helpful action [62]. The positive impact of self-enhancement on constructive thinking has been supported in numerous fields, such as knowledge sharing [65] and knowledge contribution [66]. These observations, consistent with the motive-self logic of ETM, conclude that an enhanced positive perception toward the self (self-enhancement) is a crucial incentive to activate one’s promising thinking about self-influence achievable in the surrounding SNSs (SIS). That is, people become aware of and feel confidence about their good capabilities of expertise, persuasiveness, and social connection to influence their social contacts. Overall, the preceding argument puts forth the positive relationship between self-enhancement and SIS.

**H6a:**  *Self-enhancement is positively related to SIS.*

Beyond the self-motive, social-motives may play a crucial role in inspiring SIS because they are part of egoistic motives [46], which open an avenue to one’s constructive thinking and the subsequent beneficial behaviors (e.g., volunteering [66]). According to ETM [24], social-motives (i.e., social-enhancement and response efficacy) thereupon are positioned as the coordinated motives, supplementary to the self-motive, to drive self-influence pertaining to one’s social world (i.e., SIS in our case). Social-enhancement focuses on the desire for ensuring the acceptance and approval of others and advancing one’s social status within SNSs [65], while response efficacy concerns one’s perception of the effectiveness of continuously implementing certain behaviors to help one’s surrounding environments (e.g., PEBs, green behaviors [67,68]). As scholars [65,69] indicated, in addition to self-enhancement, people get involved in social activities because they aspire to fulfill the need for enriching themselves by means of enhancing their social status (i.e., social-enhancement) on the one hand, and by an effective way to fulfill the important need and execute beneficial behaviors (i.e., response efficacy [67,70]) on the other. Given that social-enhancement and response efficacy have consistently been found to be a crucial role in inspiring people’s favorable tendency to execute various helpful behaviors (e.g., PEBs, knowledge contribution, green consumption) [65,67,68,69,70], it is justified to incorporate these two factors as social-motives in this study.

Specifically, researchers [67,68] have noticed that people devote more attention to certain pro-environmental issues once they realize the meaningfulness of their help and response to environmental sustainability (response efficacy). Zhao et al. [68] further highlights the more significant role of response efficacy than other self factors in provoking people’s coping tendencies and actions. Likewise, Chiu et al. [65] noticed that social-enhancement outperforms other factors in enriching the self and thus helps generate preference to one’s social surroundings, thus and resulting in helping behaviors. Along the same logic, we propose social-enhancement and response efficacy as motivators to encourage people to think positively about their influence achievable in their surrounding SNSs and what they can do to help make the world better. Indeed, such operationalization (i.e., treating social-enhancement and response efficacy as social-motives) not only contributes to the literature on ETM but also advances our research model because it incorporates more essential factors to complement the self-motive in enlightening SIS. Hence, the two hypotheses related to social-motives and SIS are proposed as follows:

**H6b:**  *Social-enhancement is positively related to SIS.*

**H6c:**  *Response efficacy is positively related to SIS.*

#### 3.4.2. Self-Influence on SNSs and PEBs

ETM postulates that people tend to judge themselves favorably and this positive self-view dominates the sense-making of their behaviors and actions [24]. Indeed, SIS properly exemplifies such an egocentric view of ETM when applied to our investigated context (SNSs) because it interprets one’s knowledge about the influence of self-exertion in the context of SNSs [23]. This study extends the concept of ETM and postulates that SIS might be a remarkable driver of private and public PEBs in the context of SNSs, which has not been conceptually nor empirically investigated. Although existing literature is still scarce in the proposed relationship between SIS and PEBs, studying SIS and PEBs does have its theoretical support. According to ETM [24], people’s self-concept about their social world (SIS, in our case) not only consciously responds to proximately excited motives but also determines their subsequent actions (PEBs in our study). The literature on SIS [23] also confirms the positive impact of SIS on people’s further actions (e.g., pass-along behavior; willingness to speak out [71]). Along the same logic, SIS may manipulate the perceived meanings of PEBs and inspire people’s pro-environmental actions in the context of SNSs. That is, when people feel confidence in their SNS-influence in terms of maven, persuasiveness, and connectivity, they are more likely to undertake PEBs, such as disseminating information on environmental issues and campaigns on their SNSs (public PEB [72]), and performing recycling as well as reusing behaviors (private PEB [73]). This is because they believe that devoting their abilities to influencing others (e.g., their SNS contacts) can make a meaningful difference and make the environment better. Overall, these observations provide theoretical backing to support our proposed association between SIS and both PEBs. Hence,

**H7a:**  *SIS is positively related to public PEB.*

**H7b:**  *SIS is positively related to private PEB.*

## 4. Method

### 4.1. Measures

This study applied a survey method to collect data and verify our proposed hypotheses. Items of the survey measures were primarily adapted from related literature to establish survey content validity. All items (see Appendix A) were measured with a seven-point Likert scale (1 = strongly disagree, 7 = strongly agree). Particularly, justification for treating self-influence on SNSs (SIS) and social stress as reflective-formative second-order constructs is threefold: (1) both constructs are formed by reflectively individual first-order constructs [74]; (2) this operationalization is congruent with prior studies [17,23]; and (3) this operationalization can parsimoniously illuminate complicated situations [75]. Explicitly, SIS is formed by three first-order constructs (i.e., maven, connectivity, and persuasiveness). Each of them incorporated five items [23,60] and should be clearly distinguishable, unique, and not interchangeable [74]. Likewise, in line with Maier’s [17] work, social communication overload (four items), social action overload (five items) and social information overload (six items) jointly form the second-order construct of social stress, rather than the other way around [74].

Generally, measures for awareness of consequences (five items) and ascription of responsibility (four items) were adapted from previous studies [76,77] to reflect the perceived consequences and responsibility when receiving eco-messages in Facebook. Measures of personal norms (five items; [78,79]) were focused on PEBs. A sample item is “I feel that I have an ethical/moral obligation to engage in PEBs”. Measures for public PEB (seven items) and private PEB (four items) were adapted from existing works [22,72,73,80] to specify the related PEBs in Facebook. Guilt (four items) was adapted from Lau-Gesk and Meyers-Levy [81] to denote the feeling of guilt if one does not engaging in PEBs. Measures for self-enhancement (four items [82]), social-enhancement (four items [83]), and response efficacy (five items [68]) were refined to fit the issue of PEBs in Facebook. A sample item of self-enhancement is “I feel that I have a number of good qualities by sharing eco-messages and showing my PEBs in Facebook”. A sample item of social-enhancement is “I feel that I can earn respect from other people by sharing eco-messages and showing my PEBs in Facebook”. A sample item of response efficacy is “I am confident that, together, we can save natural resources”. Draft instruments were also appraised by three professors and six graduate students familiar with the theme of PEB and SNSs. Upon their feedback, certain wording of initial items was refined to accurately convey the proper meaning of the construct.

### 4.2. Survey Administration and Sample Profile

Before the formal survey, a pretest was executed with 30 graduate students who had PEB experience on SNSs to ensure the readability of the questionnaire (e.g., assessing ease of understanding, logical consistency, and contextual relevance of the measures). After rewording inapplicable or confusing terms, the formal survey was published online. To validate our research model, we invited those users of Facebook with experience of participating in pro-environment campaigns (e.g., receiving, sharing, and posting pro-environment information) to support our survey. The first page of the survey specified the study purpose, the identity of its researchers, the length of its questionnaire, the crucial value of respondent involvement, along with the incentive and the confidential assurance of respondent participation. To validate respondent eligibility, a screen question was initially applied to eliminate those respondents who did not perform PEBs within the past three months. Then, qualified respondents were requested to revive their most impressive PEB experience over the last three months to respond the questionnaire. In addition, in order to enhance the quality of survey responses, an incentive of a prize drawing of US$250 cash was offered to qualified respondents. Ultimately, the received questionnaires were screened for completeness and usability, which resulted in 897 qualified questionnaires for subsequent data analysis.

In essence, the 897 valid questionnaires consisted of 56.4% male respondents, with an average age of 24.5 years (a standard deviation of 6.8). Of the respondents, the majority had an education level of university or higher (86.7%). All 897 respondents were currently active users of Facebook because 92% had visited Facebook over 10 times per week, and 65% had over five posts per week. The average Facebook usage tenure of our respondents was 9.6 years with a standard deviation of 2.6. Our demographics are also congruent with the profile of Facebook users reported by a 2021 survey (https://tech.azuremedia.net/2021/02/15/8460/, accessed on 6 June 2022) that the majority of Facebook users in our targeted area were between 20 and 29 years of age and male users were slightly higher than female.

## 5. Data Analysis and Results

Beyond the abovementioned expert review, certain reverse-scored items were included in the survey questionnaire to evade common method bias (e.g., ambiguity and acquiescence problems) in survey design [84]. After data collection, common method bias was examined by two means. First, the correlation matrix (Table 1) showed no constructs with high correlation (r > 0.90), meaning the model is free of common method bias [85]. Second, Harman’s one-factor test was performed and concluded that the greatest factor (10.95%) did not explain most of the variance [86]. Overall, common method bias is unlikely to be a problem for subsequent analysis. Moreover, the risk of multicollinearity was detected using variance inflation factor (VIF) and condition number tests. Our results indicated that VIF (ranging from 1.37 to 3.09) is below the threshold of 3.3 and the condition number 4.95 (√(5.38/0.22)) below 10 [87], confirming the low risk of multicollinearity in the current data.

SmartPLS 3.2.8 [88] was adopted to evaluate the research model based on the following considerations. First, partial least squares (PLS) is predictive and well-suited for analyses involving complex models (e.g., second-order and hierarchical component models [89]). Since our proposed model incorporates two reflective-formative second-order constructs (social stressor and SIS), PLS also facilitates the estimation of further information concerning their dimensions. Second, PLS makes minimal demands pertaining to sample size, residual distribution, and measurement scales [90]. A two-step approach is used for data analysis. The first step includes evaluation of the measurement model, whereas the second examines structural relationship of the model.

### 5.1. Measurement Model

Initially, this study evaluated the measurement model to ensure its reliability, convergent validity, and discriminant validity. Analysis results conclude sufficient reliability and convergent validity because Table 1 demonstrates that the whole constructs go beyond the criterion value of 0.7 for composite reliability (CR), 0.5 for average variance extracted (AVE), and 0.7 for Cronbach’s alpha (α), along with all of their indicator loadings (see Appendix A) above 0.7 [91]. Adequate discriminant validity is evident using Fornell and Larcker’s [91] guideline and the heterotrait–monotrait ratio (HTMT) method [92]. Explicitly, Table 1 exhibits the square root of each construct’s AVE above all of its correlations with other constructs [91]. Table 2 reveals the HTMT matrix values under the criterion of HTMT 0.85 and the upper confidence intervals proceeded from the HTMT bootstrapping technique under the threshold of 1 [92].

### 5.2. Structural Model

**Figure 2:**
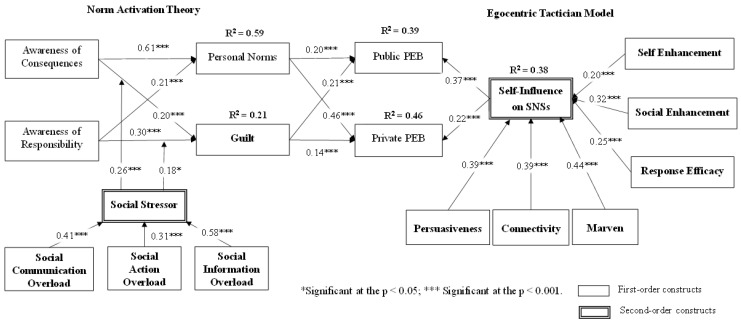
Analysis Results for the Proposed Model.

Then, this study determined the explanatory power of the structural model using the R^2^ values and structural paths of endogenous variables (see Figure 2). Based on a bootstrap procedure (1000 bootstrap runs), every path in Figure 2 was significant (*p* < 0.01), indicating full support for our proposed hypotheses. The explanatory power of our research model is validated because the R^2^ values for personal norms (0.59), guilt (0.21), public PEB (0.39), private PEB (0.46), and SIS (0.38) predict a considerable proportion of the variance. Moreover, the global validation of our PLS-oriented model is confirmed because its goodness-of-fit (Gof) index (0.52), estimated from the geometric mean of the average communality and average R^2^ (for endogenous constructs), exceeds the threshold for a great effect size (0.36) [93]. The predictive capability of our model is also empirically supported using the Stone-Geisser’s Q^2^ test [94]. The Q^2^ values, estimated with a blindfolding method, for personal norms (0.37), guilt (0.16), public PEB (0.23), private PEB (0.26), and SIS (0.47) significantly go beyond either 0.15 or 0.35, providing medium or large predictive relevance for our model [89]. In sum, the satisfactory results of R^2^, Gof, and Q^2^ verify the superior quality of our model.

## 6. Discussion

Notwithstanding SNSs being an efficient means to promote PEBs, existing literature on driving forces of PEBs has largely hinged on the perspective of NAT but not on consideration related to SNSs. This observation indicates a potential research direction to be explored since SNS-oriented notions and theories may play a crucial role in facilitating PEBs. Against this point, this study is of great importance in this direction because we devised a unifying model incorporating multidisciplinary theories (i.e., ETM and guilt) and SNS-oriented factors (social stressor and SIS) to supplement NAT in progressing PEB research on SNSs. Study results are noteworthy for further discussion, along with their academic and practical implications.

First, study findings indicate that users of SNSs do engage in both public PEB and private PEB, with a high mean of 5.5 and 4.5, respectively. This observation evinces SNSs to be an efficient way not only to distribute pro-environment campaigns but also to activate SNS users’ engagement towards PEBs. Beyond the traditional NAT factor (personal norms), this study verified the significant influence of guilt and SIS on both PEBs, supporting the related hypotheses (H4a, H4b, H7a, and H7b). Specifically, for public PEB, either guilt (β = 0.21) or SIS (β = 0.37) hold relatively stronger effects than that of personal norms (β = 0.20). Opposite results are found for private PEBs. Our findings reveal that in terms of increasing private PEB, personal norms (β = 0.46) takes the lead, followed by SIS (β = 0.22) and guilt (β = 0.14). With the evaluation of path coefficient, it can be suggested that in the context of SNSs, SIS plays a more important role than others in explaining public PEB, whereas personal norms keeps its supreme role in illuminating private PEB. These findings conclude the applicability of SIS and guilt along with associated theories (i.e., theories of ETM and emotion) in advancing traditional wisdom around NAT and PEBs, which also respond to the abovementioned second research question.

Second, study results confirm our expectation that both the complete NAT model and the proposed ETM path can account for these two types of PEBs. To evaluate the substantial value of the added ETM path, we conducted a model comparison between the comprehensive model (including both the NAT model and the ETM path; Figure 2) and a model without the ETM path to public PEB (R^2^ = 28%) and private PEB (R^2^ = 42%). The R^2^ differences (11% and 4%) between both models were applied to judge the incremental validity of containing the notions of ETM. Further significance tests with *F*-values and effect sizes indicated that both R^2^ differences are statistically significant with effect sizes *f*^2^ of 0.15 (a medium-large effect size) and 0.07 (a small-medium effect size), respectively (According to Chin et al.’s [95] guideline, effect sizes *f*^2^ of 0.02, 0.15, and 0.35 are indicated to be small, mediate, and large effects, respectively. Chin et al. [95] further advise that a small *f*^2^ does not mean an unimportant effect). Test results support the contribution of R^2^ values relating to the ETM path to public PEB and private PEB. Evidently, the comprehensive model holds relatively higher explanatory power than the one without the ETM path.

Third, regarding the ETM path, this study provides evidence that three motive factors (self-enhancement, social-enhancement, and response efficacy) are significantly related to SIS (H6a, H6b, and H6c; β = 0.20, 0.32, and 0.25, respectively) to engage in both public and private PEBs (behavior; H7a and H7b). Indeed, these three motives collectively explain 38% of the variances in SIS. Explicitly, social-motives (i.e., social-enhancement and response efficacy) hold relatively more influence than self-motive in energizing SNS users to manipulate their influence (SIS), which is consistent with Chiu et al.’s [65] results. These observations illustrate that there is added value to taking social-oriented factors into consideration and applying the motive-self-behavior framework of ETM to investigate a matter occurring in the context of SNSs.

Fourth, concerning the NAT model, our findings are in line with past literature [9,45,49]. Specifically, traditional NAT factors, awareness of consequences (β = 0.61), and ascription of responsibility (β = 0.21), remain powerful in affecting personal norms, even in the context of SNSs, thus verifying our hypotheses of H1a and H1b. Likewise, personal norms is significantly related to both public and private PEBs, thus supporting H2a and H2b. Beyond the traditional NAT path, study results show the significant contribution of the proposed emotional path to both types of PEBs in completing the NAT model. As Figure 2 shows, both conventional NAT factors, awareness of consequences (β = 0.20; H3a) and ascription of responsibility (β = 0.30; H3b), also hold significant impacts on guilt, which in turn significantly affects public and private PEBs (H4a and H4b; β = 0.21 and 0.14). A path coefficient comparison shows that guilt (β = 0.21) holds relatively more influence than personal norms (β = 0.20) on public PEB. This observation suggests that incorporating the emotional notion of guilt indeed sheds further light on the determinants of PEBs.

Fifth, this study further incorporates a moderator of social stressor along with the emotional path. Study findings reveal the significant moderating effects on associations between awareness of consequences and guilt (β = 0.26) and between ascription of responsibility and guilt (β = 0.18), thus supporting the moderating hypotheses of H5a and H5b. Furthermore, after investigating the full model (i.e., the emotional path with the interaction effects of social stressor), its R^2^ value of guilt (24%) was compared to that of a model without the moderator (21%) to judge the interaction effect [95]. Our hierarchical difference test resulted in an effect size *f*^2^ of 0.04, implying between the small and medium effect size. It is unfortunate to conclude that a small *f*^2^ is not a noteworthy effect [95]. Instead, because the full model has significantly higher explanatory power than the one without the interaction effects, social stressors indeed contribute positive effects on the relationships between awareness of consequences, ascription of responsibility, and guilt.

Sixth, a post-hoc test was performed to explore the possible impacts of demographic variables (i.e., age and gender) on our two outcome variables. Our results found no significant effects of them on private PEB (β = 0.06 and 0.05, respectively) and public PEB (β = 0.06 and 0.04, respectively).

## 7. Implications, Limitations, and Future Research

### 7.1. Theoretical Implications

This study makes four critical theoretical contributions. First, this study represents the first scholarly effort to investigate PEBs in the context of SNSs from the theories of ETM, social stressors, and guilt beyond merely the traditional view of NAT. As our literature review indicated, the majority of studies on PEBs has hinged on NAT [9,10]. However, it appears illogical to investigate the theme of PEBs in the context of SNSs without considering factors applicable to the investigated context. Against this background, this study followed Taylor and Strutton’s [96] suggestion to devise an integrated model incorporating multidisciplinary theories along with relevant factors to advance our understanding of the focal issue. To this end, this study makes a crucial step forward by (1) searching for new factors, (2) providing evidence for the superior capability of our integrated model, and (3) corresponding to Taylor and Strutton’s [96] request for cross-disciplinary research.

Second, connecting back to the NAT literature and existing works on PEBs [9,10], a crucial value of this study inheres in its expansion of the boundary of NAT. Specifically, this study empirically verifies the influences of traditional NAT factors on PEBs (i.e., the NAT path) even in the virtual context of SNSs, thus confirming the applicability of NAT from the physical context to the virtual social milieu. Indeed, centered on personal norms, the NAT path represents a positive consideration to activate people’s PEBs. To supplement the NAT path in explaining PEBs, this study incorporates an emotional path with one averse emotion of guilt to present a negative stance. Study findings empirically confirm the motivational force of guilt to trigger constructive actions (i.e., two types of PEBs in our case). Apart from being consistent with existing literature on guilt [14,30], our results also evidence the contribution of adding the negative consideration of guilt to the original NAT path that has yet been unnoticed in the literature.

Third, alongside the emotional path, this study further incorporates a social stressor as a moderator to achieve a deeper understanding of the associations between awareness of consequences, ascription of responsibility, and guilt. Generally, previous research has mostly focused on the direct effects of social stressor on several negative outcomes (e.g., SNS exhaustion, discontinuous usage intention, SNS fatigue [17,36]). Instead, our results conclude a social stressor is a helpful moderator to reinforce the strength of the emotional path to foster PEBs in the context of SNSs. In essence, this study contributes to the literature on social stress by (1) theoretically exploring its moderating effects to enable simple main effects into more insightful conditional relationships [14], and (2) empirically supporting its beneficial value to amplify the emotional path to approach PEBs. These observations imply that, similar to the effect of guilt, social stressors might hold a motivation force to facilitate positive outcomes when it is applied to a suitable matter and setting. Future studies are warranted to explore this theme.

Fourth, a significant and notable contribution of this study lies in the application of ETM to develop a ETM path along with social and SNS-oriented factors to enlighten PEBs theoretically and empirically. Study findings indicate that, in addition to personal norms, SIS (though previously omitted in the literature) might be a key driver of PEBs, particularly in virtual social environments (e.g., SNSs). Specifically, the magnitudes of the path coefficients for SIS on public PEB and for personal norms on private PEB insinuate that they are undoubtedly crucial and corresponding determinants of PEBs. In addition, based on the motive-self-behavior scheme of ETM, this study incorporates both self- and social- motives (self-enhancement, social-enhancement, and response efficacy) to constitute the ETM path. The study evidences that people indeed involve multiple considerations when forming egoistic motives in their social surroundings, which significantly awakens their attention to their influence on SNSs (SIS), and ultimately actualizes SIS by performing PEBs. To this end, this investigation also responds to Pan et al.’s [13] call for more IS research efforts to study the issue on how citizen involvement can mitigate climate-change-related impacts. Overall, this study adds novelty to the literature by opening new research horizons for PEBs and expanding the theoretical boundary of ETM to a contemporary social theme.

### 7.2. Practical Implications

The first remarkable implication for practice is that policy-makers and non-governmental organizations (NGOs) should not merely focus on traditional NAT considerations when promoting PEBs. In the light of facilitating PEBs, this study suggests that (1) utilizing SNSs should be an effective means to expedite the process, and (2) encompassing manifold thoughts (i.e., personal norms along with guilt and ETM consideration) advances our understanding of the matter. Our work evidences the extra contribution of the ETM path on fostering PEBs, supporting the merit of the egocentric tactician view. Specifically, study findings reveal that in the context of SNSs, SIS indeed holds the strongest effect on public PEB (than personal norms and guilt), while social-enhancement outperforms other motives (self-enhancement and response efficacy) in affecting SIS. From the viewpoint of policy-makers and NGOs, it would be somewhat harsh to infer our findings as saying that self-enhancement and response efficacy may deserve less attention. The proper justification is that given the investigated context of our sample, further increments in self-enhancement and response efficacy may be less potent than parallel increments in social-enhancement. These observations provide two promising directions for practitioners to work smart with state-of-the-art SNSs. First, the importance of self-enhancement and response efficacy should not be discounted because they do play a part in inspiring people to think of self-influence in their surrounding SNSs. Second, although self-enhancement cannot be easily manipulated by practitioners, social-enhancement and response efficacy are relatively more observable in the context of virtual SNSs than in the physical context because of the presented affordances of SNSs (e.g., visibility, selectivity, persistence, and interactivity [28]). This is good news for practitioners because they would be better off to make good use of the diverse techniques and mechanisms accessible in SNSs (e.g., sending notifications of events of interest, posting status updates) to enhance these motives. For example, in the context of SNS, it is relatively easier for people to notice certain social cues regarding the effectiveness of their efforts to improve the environment as well as the approval and respect from others. These positive motives would awaken their thoughts about their influence on SNSs (SIS) and eventually actualize their influence by performing both public and private PEBs.

Study results concerning the emotional path should be of special interest to practitioners. While conventional wisdom in PEB research has long emphasized NAT, our findings indicate that when determining PEBs in SNSs, the negative emotion of guilt matters because its motivational force to take reparative actions (i.e., PEBs) remains significant in our investigated theme. These observations suggest that advocating pro-environmental campaigns, policies, and eco-messages through SNSs should be a worthy direction for policy-makers and NGOs to make continuous efforts to achieve environment protection. Apparently, the advantages of SNSs (e.g., richer information, effectiveness diffusion, wider social-connections with trustworthy information) (Please see Section 2.1. “Eco-Message Diffusion and Pro-Environmental Behavior in Social Networking Sites” for details) expedite the eco-message dissemination and increase the occurrences of two activators (i.e., awareness of both consequences and responsibility), which awaken not only personal norms but also the feelings of guilt, then eventually hold better chance to energize the actions of PEBs.

Furthermore, a unique practical implication of our work lies in exploring the moderator of social stressors along the emotional path. It is worth noting that the associations between two activators and guilt can be strengthened when taking this social stressor into account. Generally, it may be true that frequently disseminating eco-messages and pro-environmental campaigns might put a social stressor on SNS users because the social overloads in terms of information, communication, and action are beyond their capabilities to control, which might bring about SNS exhaustion [18]. However, every cloud has a silver lining. This study, instead, plausibly suggests that for practitioners, with proper applications, social stressors can help reverse undesired outcomes to constructive actions by reinforcing the emotional path towards PEBs. This argument is reasonable because intensive social communications, actions, and the related excessive exposure to an eco-message indeed not only highlights the significance of the environmental issue but also draws receivers’ attention. As such, a social stressor might deepen their feelings of guilt owing to their previous inactions or wrongdoings as well as their awareness of unfavorable outcomes, and ultimately energize their PEBs privately and publicly as compensation.

### 7.3. Limitations and Future Research Directions

This study has three limitations that may pave new research avenues to be explored. First, our results may have been affected by selection bias because we constrained the study scope on purpose, merely collecting data from existing users of SNSs who have performed PEBs. Non-registered users or SNS users without PEB experiences might have different thoughts about the influence of our proposed factors (e.g., NAT factors, guilt, ETM factors, and social stressors). Therefore, study results should be merely inferred to account for the PEBs of existing SNS users. Accordingly, one interesting direction for future research is to investigate the proposed determinants of PEBs from the aspect of non-registered people. Second, because of the cross-sectional design of the current study, all of the statistically supported associations can be concluded only as tentative. Future studies are encouraged to apply longitudinal studies to validate causal relationships among our proposed constructs. Third, the present study considers merely one construct (i.e., social stressors) as a moderator along the emotional path in the first-part model. Future research could explore alternative moderators along the NAT path and especially the ETM path to uncover further thoughtful and systematic relationships.

## Figures and Tables

**Table 1:** Correlations among constructs and the square root of the AVE.

	AVE	CR	α	AC	AR	CO	RE	GU	MA	PN	PER	PRP	PUP	SAO	SCO	SOE	SEE	SIO
AC	0.64	0.90	0.86	**0.80**														
AR	0.77	0.93	0.90	0.62	**0.88**													
CO	0.79	0.95	0.93	0.12	0.14	**0.89**												
RE	0.74	0.93	0.91	0.68	0.56	0.17	**0.86**											
GU	0.76	0.93	0.89	0.40	0.44	0.28	0.41	**0.87**										
MA	0.73	0.93	0.91	0.44	0.38	0.43	0.47	0.49	**0.85**									
PN	0.67	0.91	0.88	0.65	0.63	0.17	0.68	0.50	0.50	**0.82**								
PER	0.77	0.94	0.92	0.29	0.28	0.53	0.30	0.27	0.55	0.29	**0.87**							
PRP	0.65	0.86	0.77	0.56	0.45	0.26	0.63	0.47	0.51	0.62	0.35	**0.81**						
PUP	0.66	0.93	0.91	0.36	0.33	0.35	0.38	0.47	0.62	0.45	0.32	0.49	**0.81**					
SAO	0.68	0.91	0.88	0.01	0.00	0.36	0.02	0.16	0.25	0.06	0.16	0.07	0.35	**0.83**				
SCO	0.65	0.88	0.82	0.06	0.07	0.23	0.04	0.19	0.15	0.13	0.15	0.14	0.25	0.30	**0.81**			
SOE	0.86	0.96	0.95	0.15	0.12	0.47	0.19	0.38	0.51	0.25	0.28	0.29	0.55	0.42	0.18	**0.93**		
SEE	0.90	0.97	0.96	0.29	0.24	0.42	0.39	0.44	0.58	0.40	0.30	0.41	0.61	0.35	0.15	0.64	**0.95**	
SIO	0.65	0.92	0.89	0.18	0.20	0.14	0.14	0.18	0.14	0.19	0.15	0.16	0.19	0.14	0.60	0.13	0.10	**0.81**

Note: α: Cronbachs α; CR: Composite Reliability. The diagonal elements (in bold) represent the square root of the AVE. Please refer to Table A1 for the abbreviation of each construct.

**Table 2:** Heterotrait-Monotrait Ratio (HTMT).

	AC	AR	CO	GU	MA	PER	PN	PRP	PUP	RE	SAO	SCO	SIO	SEE	SOE
AC															
AR	0.68														
CO	0.13	0.15													
GU	0.44	0.47	0.30												
MA	0.49	0.42	0.47	0.54											
PER	0.33	0.31	0.57	0.29	0.60										
PN	0.68	0.71	0.20	0.56	0.56	0.32									
PRP	0.67	0.54	0.30	0.55	0.61	0.41	0.66								
PUP	0.39	0.36	0.38	0.51	0.68	0.35	0.51	0.59							
RE	0.67	0.62	0.18	0.44	0.51	0.33	0.76	0.75	0.41						
SAO	0.06	0.04	0.39	0.19	0.28	0.18	0.09	0.09	0.39	0.06					
SCO	0.08	0.08	0.26	0.22	0.18	0.17	0.16	0.17	0.29	0.06	0.35				
SIO	0.21	0.24	0.15	0.20	0.16	0.17	0.22	0.20	0.21	0.16	0.16	0.70			
SEE	0.32	0.25	0.44	0.47	0.63	0.32	0.44	0.47	0.65	0.41	0.37	0.17	0.13		
SOE	0.16	0.13	0.50	0.42	0.55	0.30	0.29	0.34	0.59	0.20	0.46	0.21	0.15	0.67	

Note: Please refer to Table A1 for the abbreviation of each construct.

## Appendix A

**Table A1:** Summary of questionnaire items.

Construct Items		Loadings
Social action overload (SAO) [17]		
Regarding your social experience in Facebook, …		
SAO1	I take too much care of my friends’ well-being on Facebook.	0.79
SAO2	I deal too much with my friends’ problems on Facebook.	0.85
SAO3	My sense of being responsible for how much fun my friends have on Facebook is too strong.	0.82
SAO4	I am too often caring for my friends on Facebook.	0.84
SAO5	I pay too much attention to posts of my friends on Facebook.	0.82
Social communication overload (SCO) [17]		
Regarding your social communication in Facebook, …		
SCO1	I receive more messages, notifications, and announcements of nodded acquaintances on Facebook than I can respond to.	0.82
SCO2	I am overextended from the messages, notifications, and announcements I receive on Facebook.	0.87
SCO3	The amount of trivial communication on Facebook is too high.	0.80
SCO4	I receive and send too many messages, notifications, and announcements on Facebook.	0.76
Social information overload (SIO) [17]		
Regarding your social communication in Facebook,…		
SIO1	there is more information on Facebook than I can digest.	0.80
SIO2	the information on Facebook overextends me.	0.82
SIO3	it is difficult for me to focus on the essential information on Facebook.	0.88
SIO4	the amount of information on Facebook makes me overlook important information.	0.85
SIO5	I am faced with too much irrelevant information on Facebook.	0.79
SIO6	I do not receive too much information on Facebook. (R)	0.80
Ascription of responsibility (AR) [76]		
Upon receiving eco-messages in Facebook, what do you feel about your responsibility?		
AR1	I take joint responsibility for the depletion of energy resources.	0.90
AR2	I feel jointly responsible for the greenhouse effect.	0.91
AR3	I do not take joint responsibility for environmental problems. (R)	0.92
AR4	My behavior that is not eco-sensitive contributes to environmental problems.	0.80
Awareness of consequences (AC) [76,77]		
Upon receiving eco-messages in Facebook, what do you feel about the consequences of environment problems?		
AC1	The greenhouse effect is a problem for society.	0.80
AC2	Energy conservation contributes to a reduction of the greenhouse effect.	0.81
AC3	I am aware of the impact of non-PEB (e.g., no recycling) on our society or environment.	0.78
AC4	I try to reduce energy consumption.	0.82
AC5	I am concerned about soil, water, and air pollution.	0.80
Personal norms (PN) [78,79]		
PN1	I personally feel I should undertake PEBs.	0.82
PN2	PEBs would not be the right thing for me to do. (R)	0.87
PN3	I feel that I have an ethical/moral obligation to engage in PEBs.	0.88
PN4	I make myself valuable when I do engage in PEB.	0.80
PN5	I would consider myself a better person if I engaged in PEBs.	0.80
Public PEB (PUP) [22,72,80]		
In Facebook, I have…		
PUP1	shared posts about certain environmental issues.	0.80
PUP2	attended environmental demonstrations about an environmental issue.	0.78
PUP3	taken part in volunteer activities for environmental conservation.	0.80
PUP4	encouraged personal contacts (e.g., friends, relatives, colleagues, or classmates) to save resources.	0.82
PUP5	encouraged personal contacts to participate in environmental activities (e.g., tree-planting, picking-up litter).	0.86
PUP6	not encouraged personal contacts to support environmental policies. (R)	0.86
PUP7	discussed environmental issues with others.	0.78
Private PEB (PRP) [72,73]		
In Facebook, I have shown my personal PEBs such as…		
PRP1	Reusing or recycling something rather than throwing it away.	0.80
PRP2	Trying to reduce water consumption.	0.82
PRP3	Avoiding using one-off chopsticks.	0.80
PRP4	Buying organic or chemical-free vegetables.	0.76
Guilt (GU) [81]		
Please indicate how common the feeling is for you if you do not engage in any pro-environmental actions when you receive a posting in Facebook.		
GU1	Guilty.	0.92
GU2	Repentant.	0.93
GU3	Blameworthy.	0.85
GU4	Responsible.	0.80
Maven (MA) [23,60]		
In the context of Facebook, …		
MA1	when I know something about an environmental news item, I feel it is important to share that information with others.	0.83
MA2	I like to be aware of the most up-to-date environmental news so I can help others by sharing when it is relevant.	0.89
MA3	If someone asked me about an environmental issue that I was unsure of, I would know how to help them find the answer.	0.84
MA4	Being knowledgeable enough about an environmental issue so that I could teach someone else is important to me	0.89
MA5	People often seek me out for answers when they have questions about an environmental issue.	0.81
Persuasiveness (PER) [23,60]		
In the context of Facebook, …		
PER1	I am good at thinking of multiple ways to explain my position on an issue.	0.85
PER2	When in a discussion, I’m able to make others see my side of the issue.	0.87
PER3	I am able to adapt my method of argument to persuade someone.	0.89
PER4	I can effortlessly offer multiple perspectives on an issue which support my position.	0.89
PER5	More often than not, I am able to convince others of my position during an argument.	0.88
Connectivity (CO) [23,60]		
In the context of Facebook, …		
CO1	I’m often the link between friends in different groups.	0.88
CO2	I often find myself introducing people to each other.	0.92
CO3	I try to bring people I know together when I think they would find each other interesting.	0.85
CO4	I frequently find that I am the connection between people who would not otherwise know one another.	0.91
CO5	The people I know often know each other because of me.	0.91
Self-enhancement (SEE) [82]		
By sharing eco-messages and showing my PEBs in Facebook, I feel that…		
SEE1	I have a number of good qualities.	0.95
SEE2	I am able to do things as well as most other people.	0.95
SEE3	I am not a person of worth, at least on an equal plane with others. (R)	0.95
SEE4	I am competent.	0.94
Social-enhancement (SOE) [83]		
By sharing eco-messages and showing my PEBs in Facebook, I feel that…		
SOE1	I can earn respect from other people.	0.92
SOE2	my status in the profession can be enhanced.	0.94
SOE3	my reputation in the profession cannot be enhanced. (R)	0.94
SOE4	I can gain approval from other friends.	0.92
Response efficacy (RE) [68]		
RE1	I am sure that our environmentally friendly behaviors can have a positive effect on the environment.	0.84
RE2	I am confident that, together, we can save natural resources.	0.88
RE3	We can do nothing to help control pollution of the environment. (R)	0.82
RE4	It is worth it for every individual to make efforts together to preserve and improve the environment.	0.86
RE5	Since each individual can have any effect upon environmental problems, what we do can make a meaningful difference.	0.88

Note: R, reverse-coded.
